# A Note on the Treatment of Migraine

**Published:** 1889-03

**Authors:** T. J. Bokenham

**Affiliations:** St. Bartholomew’s Hospital


### A NOTE ON THE TREATMENT OF MIGRAINE BY ANTIPYRIN.

BY T. J. BOKENHAM, L. S. A., ST. BARTHOLOMEW'S HOSPITAL.

Antipyrin has for the past few months attracted considerable attention in Germany on account of its astonishing action on the paroxysms of migraine. In England, however, until quite recently, the drug does not appear to have been much used in this disease. When it has been tried at all, the doses given have been large, and have in some instances been found to produce unpleasant effects. During the last two months I have had an opportunity of treating as many as twenty-six cases of migraine with antipyrin. The results have been in every case, perfectly satisfactory, although the dose given has been quite small, in no case exceeding four grains.

It will not be necessary to detail fully each of the cases, as they all resemble one another very closely in their main features. I will only describe three typical cases: namely, that of a near relative; that of a girl, members of whose family exhibit epileptic tendencies; and an attack as occurring in myself.

My own migraine is usually brought on by over-use of the eyes, and is almost invariably preceded by well-marked teichopsia, which lasts for from half-an-hour to several hours. As ths attack proceeds, I get a tender spot on some part of the scalp, generally on the right parietal region, a vascular disturbance, with throbbing behind the right eye. The first time I took antipyrin it was in a dose of three grains after the pain was well developed. In a very short time the throbbing entirely ceased, leaving only a dull aching pain behind the eye; the flushing of the face also diminished. After half-an-hour I took a second dose of three grains, and by the end of an hour from the time of taking the first dose was quite well, save for slight tendernes of the scalp. Since then I have tried the drug, in the same dose, taking it immediately the teichopsia comes on, with complete success in altogether preventing the attack.

The second case is that of a lady who has been subject to attacks of migraine for many years. The starting point of her attacks is also in the eyes. For a long time large doses of ammonium bromide were successful in cutting short the attacks, but recently this drug seems to have lost most of its power. Antipyrin entirely relieved the prostration and pain after the second dose of three grains.

The third case is interesting in that it points to another class of cases in which antipyrin may be of use, namely epilepsy. Here the mother is epileptic, the father is of a very excitable temperament, and another member of the family shows signs of weak intellect. In this case migraine usually follows any undue fatigue, and is attended with a good deal of prostration. I happened to be with her during one attack and promptly adminisiered antipyrin. The headache yielded entirely after the second dose of four grains.

I have under observation also two epileptics in whom the fits are preceded by a well-marked aura in the shape of tinkling of the extremities of the fingers. They are directed to take, immediately they feel this sensation, ten grains of antipyrin in a little water. If the theory of Dr. Liveing concerning the close pathological relation between migraine and the other so-called paroxysmal neuroses be true, there is some hope that in both these diseases we may be able to check or to cut short the attack by the timely use of antipyrin. I have not yet had the opportunity of observing the effect on these two epileptics, but it would certainly seem to be worth a trial.

In conclusion, I beg to point out again that the plan I have pursued with so much success in this series of cases is to give very small doses, contrary to the practice of most physicians, who have used as much as fifteen grains repeated every twenty minutes. I feel sure that antipyrin requires only to be known to become a regular remedy in the treatment of migraine, and that practitioners will use the small dose with less hesitation than they would the large ones previously given.—*Practitioner*, *Feb.*, 1888, *p.* 99.

